# The importance of antioxidants which play the role in cellular response against oxidative/nitrosative stress: current state

**DOI:** 10.1186/s12937-016-0186-5

**Published:** 2016-07-25

**Authors:** Ergul Belge Kurutas

**Affiliations:** Department of Medical Biochemistry, Faculty of Medicine, Sutcu Imam University, Avsar Campus, Kahramanmaras, 46050 Turkey

**Keywords:** Antioxidants, Oxidative/Nitrosative stress, Reactive oxygen and reactive nitrogen species

## Abstract

Remarkable interest has risen in the idea that oxidative/nitrosative stress is mediated in the etiology of numerous human diseases. Oxidative/Nitrosative stress is the result of an disequilibrium in oxidant/antioxidant which reveals from continuous increase of Reactive Oxygen and Reactive Nitrogen Species production. The aim of this review is to emphasize with current information the importance of antioxidants which play the role in cellular responce against oxidative/nitrosative stress, which would be helpful in enhancing the knowledge of any biochemist, pathophysiologist, or medical personnel regarding this important issue. Products of lipid peroxidation have commonly been used as biomarkers of oxidative/nitrosative stress damage. Lipid peroxidation generates a variety of relatively stable decomposition end products, mainly α, β-unsaturated reactive aldehydes, such as malondialdehyde, 4-hydroxy-2-nonenal, 2-propenal (acrolein) and isoprostanes, which can be measured in plasma and urine as an indirect index of oxidative/nitrosative stress. Antioxidants are exogenous or endogenous molecules that mitigate any form of oxidative/nitrosative stress or its consequences. They may act from directly scavenging free radicals to increasing antioxidative defences. Antioxidant deficiencies can develop as a result of decreased antioxidant intake, synthesis of endogenous enzymes or increased antioxidant utilization. Antioxidant supplementation has become an increasingly popular practice to maintain optimal body function. However, antoxidants exhibit pro-oxidant activity depending on the specific set of conditions. Of particular importance are their dosage and redox conditions in the cell.

## Background

The human body has several mechanisms to counteract oxidative/nitrosative stress by producing antioxidants. A shift in the balance between oxidants and antioxidants in favor of oxidants is termed as “oxidative/nitrosative stress”. Paradoxically, there is a large body of research demonstrating the general effect of oxidative/nitrosative stress on signaling pathways, less is known about the initial and direct regulation of signaling molecules by ROS/RNS. Despite the assumption that antioxidants must exert beneficial effects against oxidative/nitrosative stress, many large-scale randomized controlled trials gave inconsistent and disappointing results on the prevention of chronic diseases. It is now generally accepted that there is no evidence to support the use of non-discriminative antioxidant supplements for prevention of diseases. On the other hand, recent data show that antioxidants may be effective in the prevention and/or treatment of diseases when the right antioxidant is given to the right subject at the right time for the right duration. Free radical-mediated lipid peroxidation products which, in contrast to enzymatic oxidation products, are produced by non-specific mechanisms cause oxidative/nitrosative damage, but may also induce adaptive response to enhance the expression of antioxidant enzymes and compounds. This has raised a question if removal of too many ROS/RNS by supplementation of antioxidants may upset the cell signaling pathways and actually increase the risk of chronic diseases. However, it is unlikely that antioxidants impair physiologically essential signaling pathways.

## Reactive oxygen species and Reactive nitrogen species

Free radicals are defined as “any chemical species capable of independent existence that contains one or more unpaired electrons”. Reactive oxygen species (ROS) and Reactive nitrogen species (RNS) are free radicals which are associated with the oxygen atom (O) or their equivalents and have stronger reactivity with other molecules, rather than with O_2_. Generally, ROS/RNS are generated as by-products of cellular metabolism and ionizing radiation, usually indicating the following four species: superoxide anion (O_2_^-^), hydrogen peroxide (H_2_O_2_), hydroxyl radical (OH), and singlet oxygen (^1^O_2_). The other biologically important free radicals exist: lipid hydroperoxide (ROOH), lipid peroxyl radical (ROO), and lipid alkoxyl radical (RO), which are associated with membrane lipids; nitric oxide (NO), nitrogen dioxide (NO_2_) and peroxynitrite (ONOO-), which are reactive nitrogen species; and thiol radical (RS), which has an unpaired electron on the sulfur atom [1, 2].

The most important free radicals in many disease states are oxygen derivatives, particularly superoxide anion and the hydroxyl radical. Radical formation in the body occurs via several mechanisms, involving both endogenous and environmental factors. Superoxide anion is produced by the addition of a single electron to oxygen, and several mechanisms exist by which superoxide can be produced in vivo [3]. Some molecules such as flavine nucleotides and thiol compounds are oxidized in the presence of oxygen to produce superoxide, and these reactions greatly accelerated by the presence of transition metals such as iron or copper. The electron transport chain in the inner mitochondrial membrane performs the reduction of oxygen to water. During this process free radical intermediates are generated, which are generally tightly bound to the components of the transport chain. However, there is a constant leak of a few electrons into the mitochondrial matrix and this results in the formation of superoxide [4, 5]. There may also be continuous production of superoxide anion by vascular endothelium to neutralise nitric oxide, production of superoxide by other cells to regulate cell growth and differentiation, and the production of superoxide by phagocytic cells during the oxidative burst [6, 7].

Any biological system generating superoxide anion also occurs hydrogen peroxide as a result of a spontaneous dismutation reaction. In addition, some enzymatic reactions may produce hydrogen peroxide directly [8]. Hydrogen peroxide itself is not a free radical as it does not contain any unpaired electrons. However, it is a precursor to certain radical species such as peroxyl radical, hydroxyl radical, and superoxide. Its most vital property is the ability to cross cell membranes freely, which superoxide generally can not do. Hence, hydrogen peroxide generated in one location might diffuse a considerable distance before decomposing to yield the highly reactive hydroxyl radical, which is likely to mediate most of the toxic effects ascribed to hydrogen peroxide. Hydrogen peroxide acts as a conduit to transmit free radical induced damage across cell compartments and between cells. In the presence of hydrogen peroxide, myeloperoxidase will produce hypochlorous acid and singlet oxygen, a reaction that plays an important role in the killing of bacteria by phagocytes. Cytochrome P450 (CYP450) is a source of ROS. Through the induction of CYP450, the possibility for the production of ROS, in particular, superoxide anion and hydrogen peroxide, emerges following the breakdown or uncoupling of the CYP450 catalytic cycle. Increasing evidence has indicated that numerous drugs are metabolized by multiple activated oxygen species generated in the CYP450 catalytic cycle [9]. The hydroxyl radical or a closely related species, is probably the final mediator of most free radical induced tissue damage [10]. All of the ROS described above exert most of their pathological effects by giving rise to hydroxyl radical formation. The reason for this is that the hydroxyl radical reacts, with extremely high rate constants, with almost every type of molecule found in living cells such as lipids and nucleotides. Although hydroxyl radical formation can occur in several ways, by far the most important mechanism in vivo is likely to be the transition metal catalysed decomposition of superoxide anion and hydrogen peroxide [11]. All of elements in the first row of the d-block of the periodic table are classified as transition metals. Normally, they contain one or more unpaired electrons and are hence themselves radicals when in the elemental state. However, their main feature from the point of view of free radical biology is their inconstant valence, which allows them to undergo reactions involving the transfer of a single electron [12].

The most important transition metals in various human disease are iron and copper. These elements play a pivotal role in the production of hydroxyl radicals in vivo. Hydrogen peroxide reacts with iron II (or copper I) to generate the hydroxyl radical, a reaction first described by Fenton.

$$ \mathrm{F}{\mathrm{e}}^{2+} + {\mathrm{H}}_2{\mathrm{O}}_2\to \mathrm{F}{\mathrm{e}}^{3+} + \mathrm{O}{\mathrm{H}}^{.} + \mathrm{O}{\mathrm{H}}^{-} $$

This reaction occur in vivo, but the situation is complexed by the fact that superoxide anion (the main source of hydrogen peroxide in vivo) normally also be present [13]. Superoxide anion and hydrogen peroxide react together directly to produce the hydroxyl radical, but the rate constant for this reaction in aqueous solution is actually zero. However, if transition metal ions are present a reaction sequence is established that can proceed at a rapid rate:

$$ \begin{array}{l}\mathrm{F}{\mathrm{e}}^{3+} + {{\mathrm{O}}_2}^{-}\to \mathrm{F}{\mathrm{e}}^{2+} + {\mathrm{O}}_2\\ {}\mathrm{F}{\mathrm{e}}^{2+} + {\mathrm{H}}_2{\mathrm{O}}_2\to \mathrm{F}{\mathrm{e}}^{3+} + \mathrm{O}{\mathrm{H}}^{.} + \mathrm{O}{\mathrm{H}}^{-}\end{array} $$

net result:

$$ {{\mathrm{O}}_2}^{-} + {\mathrm{H}}_2{\mathrm{O}}_2\to \mathrm{O}{\mathrm{H}}^{-} + \mathrm{O}{\mathrm{H}}^{.} + {\mathrm{O}}_2 $$

The net result of the reaction series illustrated above is known as the Haber-Weiss reaction. Although most iron and copper in the body are secluded in forms that are not available to catalyse this reaction sequence, it is still of importance as a mechanism for the formation of the hydroxyl radical in vivo. Such conditions are found in areas of active inflammation and various pathologic situations such as stroke, septic shock, ischaemia-reperfusion injury, and it is therefore likely that hydroxyl radicals contribute to tissue damage in these settings. Iron is released from ferritin by reducing agents including ascorbate and superoxide itself, and hydrogen peroxide can release iron from a range of haem proteins. Therefore, although the iron binding proteins effectively chelate iron and prevent any appreciable redox effects under normal physiological conditions, this protection can break down in disease states. The role of copper is analogous to that described above for iron [14–17].

**Fig. 1:**
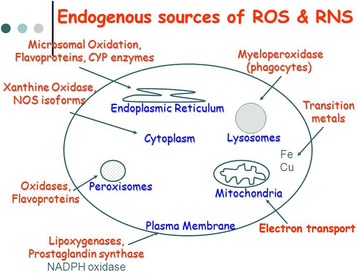
Endogenous sources of reactive oxygen and reactive nitrogen species (ROS/RNS)

Nitric oxide (NO^.^) is a small molecule that contains one unpaired electron on the antibonding 2_TTY*_ orbital and is, therefore, a radical. NO^.^ is generated in biological tissues by specific nitric oxide synthases (NOSs), which metabolise arginine to citrulline with the formation of NO^.^ via a five electron oxidative reaction [18]. NO^.^ acts as an important oxidative biological signalling molecule in a large variety of diverse physiological processes, including neurotransmission, blood pressure regulation, defence mechanisms, smooth muscle relaxation and immune regulation [19]. NO^.^ has a half-life of only a few seconds in an aqueous environment. NO^.^ has greater stability in an environment with a lower oxygen concentration (half life >15 s). However, since it is soluble in both aqueous and lipid media, it readily diffuses through the cytoplasm and plasma membranes [20]. NO^.^ has effects on neuronal transmission as well as on synaptic plasticity in the central nervous system. In the extracellular milieu, NO^.^ reacts with oxygen and water to form nitrate and nitrite anions. An important route of NO^.^ degradation is the rapid reaction with superoxide anion to form the more reactive product, peroxynitrite (ONOO^–^). Peroxynitrite reacts with proteins to form nitrotyrosine (3-NT) [21]. Immune cells, including macrophages and neutrophils, simultaneously release NO^.^ and superoxide into phagocytic vacuoles as a means of generating peroxynitrite to kill endocytosed bacteria [22]. Other inflammatory cells can also produce reactive chemicals that can result in 3-NT formation, including the peroxidases in activated neutrophils and eosinophils. 3-NT is a characteristic marker of nitrosative stress and, commonly, inflammation. Increased levels of NO and 3-NT have been reported in a variety of human skin diseases such as skin cancers, systemic lupus erythematosus, psoriasis, urticaria, and atopic dermatitis [22]. Endogenous sources of ROS/RNS are summarized in Fig. 1.

## Oxidative/nitrosative stress

Oxidative/nitrosative stress is a “privilege” of aerobic organisms. It consists by endogenous and exogenous factors. Most frequently, it is described by the production of ROS/RNS. Oxidative/nitrosative stress is a deleterious process which can be an important mediator of damage to cell structures, including lipids and membranes, proteins and DNA. However, ROS/RNS are “two-faced” products [2]. Produced in low/moderate levels as molecular signals that regulate a series of physiological processes, such as a defence against infectious agents, the maintenance of vascular tone, the control of ventilation and erythropoietin production, and signal transduction from membrane receptors in various physiological processes [23]. Many of ROS/RNS-mediated responses protect cells against oxidative/nitrosative stress and maintain “redox homeostasis”. Then, both reactive species are produced by strictly regulated enzymes, such as nitric oxide synthase (NOS), and isoforms of NADPH oxidase, or as by-products from not so well regulated sources, such as the mitochondrial electron-transport chain. Increased oxidative/nitrosative stress usually describes a condition in which antioxidant defenses are inadequate to completely inactivate ROS/RNS produced because of excessive production of ROS/RNS, loss of antioxidant defenses, or both. A major consequence of oxidative/nitrosative stress is damage to nucleic acids, lipids, and proteins, which can severely compromise cell health and viability or induce a variety of cellular responses through generation of secondary reactive species, finally causing to cell death by necrosis or apoptosis [2, 24]. Oxidative/nitrosative damage of any of these biomolecules, if unchecked, can theoretically contribute to disease development. Actually, an increasing amount of evidence shows that oxidative/nitrosative stress is connected to either the primary or secondary pathophysiological mechanisms of multiple acute and chronic human diseases [25].

Oxidative/nitrosative stress-induced peroxidation of membrane lipids can be very damaging because it leads to alterations in the biological properties of the membrane, such as the degree of fluidity, and can lead to inactivation of membrane-bound receptors or enzymes, which in turn may impair normal cellular function and increase tissue permeability. Moreover, lipid peroxidation may contribute to and amplify cellular damage resulting from generation of oxidized products, some of which are chemically reactive and covalently modify critical macromolecules [26]. Products of lipid peroxidation have therefore commonly been used as biomarkers of oxidative/nitrosative stress/damage. Lipid peroxidation generates a variety of relatively stable decomposition end products, mainly α, β-unsaturated reactive aldehydes, such as malondialdehyde (MDA), 4-hydroxy-2-nonenal (HNE), 2-propenal (acrolein) and isoprostanes (F_2_-IsoPs) [27, 28], which can then be measured in plasma and urine as an indirect index of oxidative/nitrosative stress. Compared with free radicals, the aldehydes are relatively stable and can diffuse within or even escape from the cell and attack targets far from the site of the original event. They therefore are not only end products and remnants of lipid peroxidation processes but also may act as “second cytotoxic messengers” for the primary reactions [2]. Some of these aldehydes have been shown to exhibit facile reactivity with various biomolecules, including proteins, DNA, and phospholipids, generating stable products at the end of a series of reactions that are thought to contribute to the pathogenesis of many diseases. Modification of amino acids by α, β-unsaturated aldehydes occurs mainly on the nucleophilic residues Cys and, to a lesser extent, His and Lys [29, 30]. Lipid hydroperoxides and aldehydes can also be absorbed from the diet and then excreted in urine. It follows that measurements of hydroxy fatty acids in plasma total lipids as well as plasma or urinary MDA and HNE can be confounded by diet and should not be used as an index of whole-body lipid peroxidation unless diet is strictly controlled [31]. At present, measurement of F_2_-IsoPs is regarded as one of the most reliable approaches for the assessment of oxidative/nitrosative stress status or free-radical–mediated lipid peroxidation in vivo. The current data indicate that quantification of F_2_-IsoPs in either plasma or urine gives a highly precise and accurate index of oxidative/nitrosative stress [32–34]. Now, the biomarkers of oxidative/nitrosative stress/damage and the methods used to measure them to determine an individual’s oxidative status in relation to disease conditions frequently vary among studies, making comparisons of study findings difficult. Furthermore, the validity of many biomarkers remains to be established. Assays that have been developed have several shortcomings related to *(a)* the limited specificity of the assay itself for the product of oxidative/nitrosative damage being measured; *(b)* the fact that the analyte being measured is not a specific product of a specific ROS/RNS; *(c)* the lack of sufficient sensitivity to detect concentrations of the product being measured in healthy individuals, thus not allowing the definition of a reference interval; *(d)* concentrations of the product being measured being influenced by external factors such as the lipid content of the diet; or *(e)* the assay being too invasive for in vivo investigations in humans [35]. Recently, the identification of microRNAs as biomarkers of oxidative/nitrosative damage, if validated, may open the way for the development of early detection and prevention strategies for oxidative/nitrosative stress-associated diseases [36].

## Mechanisms of cell signaling mediated by ROS/RNS

**Fig. 2:**
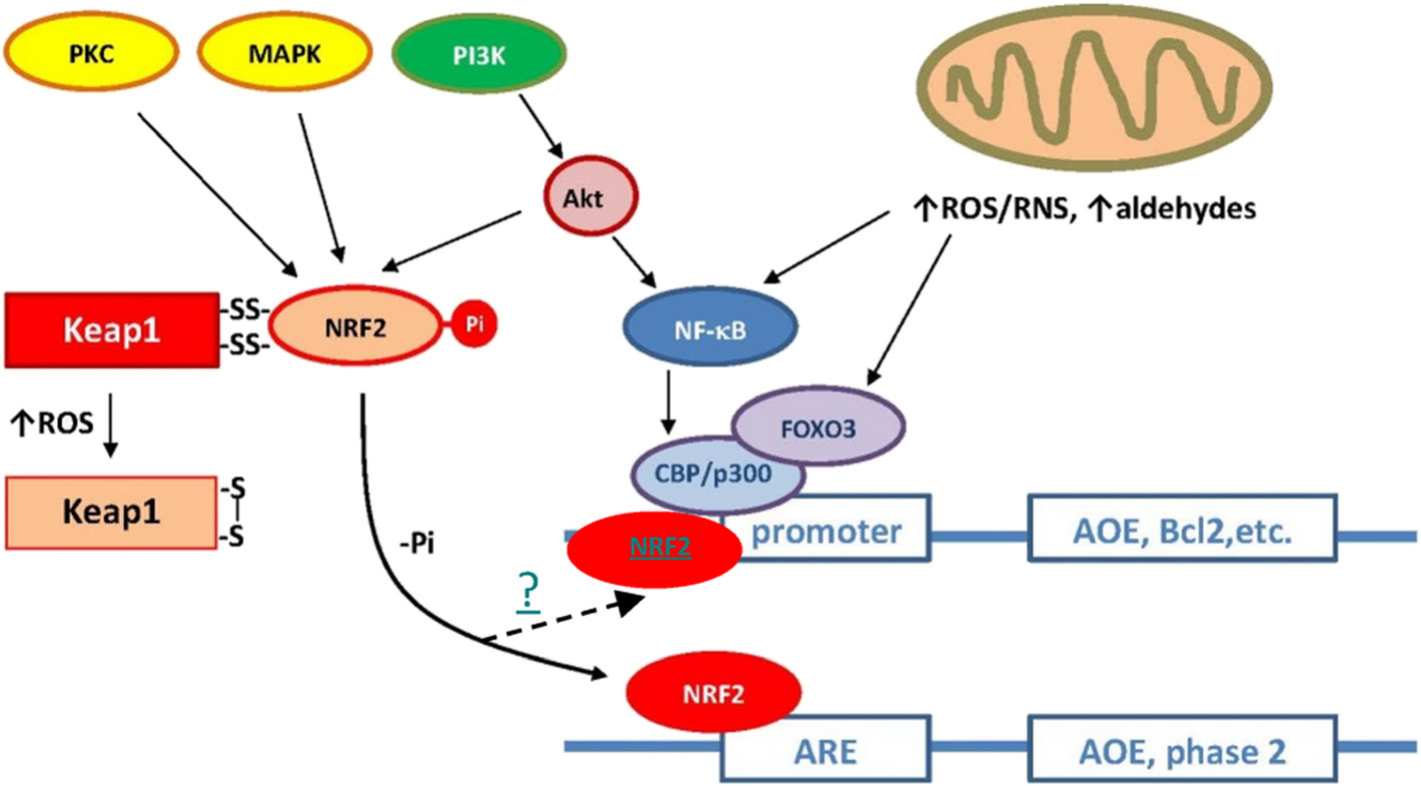
Some cellular signaling pathways in mammals. Under normal conditions (elevated intracellular reduced potential), nuclear factor erythroid 2-related factor 2 (Nrf2) is stabilized through binding to Keap-1 in the cytoplasm. Under oxidative/nitrosative stress, thiol groups in Keap-1 are oxidized (e.g., S-S cross-links) causing the dissociation of Nrf2, translocation to the nucleus, and binding to the antioxidant-responsive elements (ARE). Depending upon the binding site present in the promoter region, different antioxidant genes are induced

Cells communicate with each other and respond to extracellular stimuli through biological mechanisms called cell signalling or signal transduction. Signal transduction is a process enabling information to be transmitted from the outside of a cell to various functional elements inside the cell [37]. A biochemical basis for transducing extracellular signals into an intracellular event has long been the subject of enormous interest. Being initiators, transmitters, or modifiers of cellular response, free radicals occupy a significant place in the complex system of transmitting information along the cell to the target sensor. The effects of most extracellular signals are promoted via receptor ligation on either cell surface or cytoplasmic receptors. However, some low-molecular-weight signaling molecules, such as ROS/RNS, are able to penetrate the plasma membrane and directly modulate the activity of catalytic domain of transmembrane receptors or cytoplasmic signal transducing enzymes, thus leading to abnormal activation of transcription factors. By the initiation of gene expression and the consequent synthesis of responding functional and structural proteins, ROS/RNS allow for adaptation and survival of the cell or, depending on the intensity and duration of the signal, activate the processes responsible for the cell damage or death [38, 39]. In a given signaling protein, oxidative attack induces either a loss of function or a gain of function or a switch to a different function. The effect of ROS/RNS on the process of cell signaling is promoted through a number of simultaneous mechanisms and, most commonly, by activating an extensive network of various interactive intracellular signal transduction pathways (Fig. 2). The ability of oxidants to act as second messengers is a significant aspect of their physiological activity. The incorporation of free radicals into a complex cascade of transducing the signal to the effectors modifies and alters the order of events: numerous second messengers acquire the properties of third messengers, while intermediaries of free radical activity often function in both initiating and terminating signal transduction. These sequential events ultimately lead to either normal cell proliferation or development of cancer inflammatory conditions, aging, and two common agerelated diseases – diabetes mellitus and atherosclerosis [40–43].

## Regulation of transcription of oxidative/nitrosative stress-inducible genes – direct activation of transcription factors by oxidants

In order to prevent oxidative/nitrosative stress, the cell must respond to ROS/RNS by mounting an antioxidant defence system. Antioxidant enzymes play a major role in reducing ROS/RNS levels; therefore, redox regulation of transcription factors is significant in determining gene expression profile and cellular response to oxidative/nitrosative stress. Many hydrogen peroxide sensors and pathways are triggered converging in the regulation of transcription factors including AP-1, Nrf2, CREB, HSF1, HIF-1, TP53, NF-kB, Notch, SP1 and CREB-1, which induce the expression of a number of genes, including those required for the detoxification of oxidizing molecules and for the repair and maintenance of cellular homeostasis, controlling multiple cellular functions like cell proliferation, differentiation and apoptosis. In addition, the family of FoxO-related transcription factors plays an important role in redox responses. Hydrogen peroxide-mediated regulation of transcription factors can take place at different levels: synthesis/degradation, cytoplasm-nuclear trafficking, DNA binding and transactivation [44]. In this section we focus on the most important the activation of transcription factors by oxidative/nitrosative stress.

### Activation of Nrf2 and ARE transcription factors by ROS/RNS

Activation of the transcriptional factor called nuclear factor erythroid-derived 2-related factor 2 (Nrf2), which enhances the levels of antioxidant enzymes and phase-2-detoxifying enzymes by complex mechanisms, may be one of the ways to reduce oxidative/nitrosative stress and chronic inflammation. Antioxidant enzymes destroy free radicals by catalysis, whereas phase-2-detoxifying enzymes remove potential carcinogens by converting them to harmless compounds for elimination from the body [45]. However, increasing the levels of antioxidant enzymes by activating Nrf2 may not be sufficient to decrease oxidative/nitrosative stress and chronic inflammation optimally, because antioxidants, which are decreased in a high oxidative environment, must also be elevated. Recently, it was reported that Nrf2 provides a new therapeutic target for treatment of diabetic retinopathy and acetaminophen-induced liver injury [45, 46].

The regulation of activation of Nrf2 and proposes a hypothesis that an elevation of the levels of antioxidant enzymes and dietary and endogenous antioxidant chemicals simultaneously may reduce the incidence of cancer by decreasing oxidative/nitrosative stress and chronic inflammation. The levels of antioxidant can be increased by supplementation, but increasing the levels of antioxidant enzymes requires activation of Nrf2 by ROS/RNS-dependent and independent mechanisms [47].

The function of Nrf2 and its downstream proteins has been shown to be important for protection against oxidative/nitrosative stress- or chemical-induced cellular damage in liver [48, 49] and lung [50] as well as for prevention of cancer formation in the gastrointestinal tract [51, 52] and promotion of the wound-healing process [53]. In addition, many chronic neurodegenerative diseases (i.e. Parkinson's disease and Alzheimer's disease) and diabetic retinopathy are thought to involve oxidative/nitrosative stress as a component contributing to the progression of the disease. The regulation and cell-specific expression of these genes in cells derived from brain could therefore be important for understanding how to protect neural cells from oxidative/nitrosative stress.

The antioxidant responsive element (ARE) is a cis-acting regulatory element in promoter regions of several genes encoding phase II detoxification enzymes and antioxidant proteins [54]. The ARE plays an important role in transcriptional activation of downstream genes such as NAD(P)H:quinone oxidoreductase (NQO1), glutathione S-transferases (GSTs), glutamate-cysteine ligase (previously known as γ-glutamylcysteine synthetase), heme oxygenase-1 (HO-1), thioredoxin reductase-1 (TXNRD1), thioredoxin, and ferritin [55–59]. The DNA binding sequence of Nrf2 (5′-TGA(C/G)TCA-3′) [60] is very similar to the ARE core sequence (5′-TGACnnnGC-3′) [54]. Several lines of evidence suggest that Nrf2 binds to the ARE sequence, leading to transcriptional activation of downstream genes encoding GSTs [61–64], glutamate-cysteine ligase [65], HO-1 [63–66], and thioredoxin [59]. Previously, it was demonstrated that Nrf2 is a critical transcription factor for both basal and induced levels of NQO1 expression in IMR-32 human neuroblastoma cells [55, 56]. In contrast to the clear evidences for a role of Nrf2 in ARE activation, the upstream signaling pathway is controversial. For example, mitogen-activated protein kinase [67], protein kinase c [68], and phosphatidylinositol 3-kinase [69–72] have been suggested to play an important role in ARE activation. A recent study identified the ARE-driven genes including NQO1 that are responsible for protecting IMR-32 human neuroblastoma cells from hydrogen peroxide-induced apoptosis [69, 70] Therefore, Nrf2, which mediates transcription of ARE-driven genes, is presumably the driving force behind increasing a cluster of protective genes that play an important role in cellular defense against oxidative/nitrosative stress [73].

Keap1 (Kelch-like ECH-associated protein 1), an adaptor subunit of Cullin 3-based E3 ubiquitin ligase, regulates Nrf2 activity. Keap1 also acts as a sensor for oxidative/nitrosative and electrophilic stresses. Keap1 retains multiple sensor cysteine residues that detect various stress stimuli [74]. Post-translational modifications at the level of Keap1 that prevent its interaction with Nrf2 are another mechanism leading to Nrf2 activation. Indeed, Keap1 phosphorylation at Tyr141 renders the protein highly stable and its dephosphorylation induced by hydrogen peroxide results in rapid Keap1 degradation and Nrf2 activation [63–65]. Studies challenging the molecular basis of the Keap1-Nrf2 system functions are now critically important to improve translational studies of the system. Indeed, recent studies identified cross talk between Nrf2 and other signaling pathways, which provides new insights into the mechanisms by which the Keap1-Nrf2 system serves as a potent regulator of our health and disease [74].

Many studies have confirmed that the Keap1/Nrf2/ARE redox-sensitive signaling system plays the central role in cellular mechanisms of protection against oxidative/nitrosative stress [75]. Inducers of this system have been revealed in different studies [76]. Studies [75, 77] showed that under physiological conditions SkQ1 (10-(6′-plastoquinonyl) decyl-triphenyl-phosphonium) is a positive mediator of transcriptional activity of Nrf2 and Nrf2-controlled genes encoding antioxidant enzymes, which enhances the antioxidant potential of leukocytes in normoxia. The protective effect of SkQ1 in hyperbaric oxygen-induced oxidative/nitrosative stress might be realized via direct antioxidant properties or indirectly by stimulation of the Keap1/Nrf2/ARE signaling system. The administration of mitochondria-targeted antioxidant and changes in expression profiles of Nrf2 and Nrf2-controlled genes encoding antioxidant enzymes occur together with changes in their activity in the blood leukocytes of rats in hyperoxia. Under these conditions, the activity of superoxide dismutase and glutathione-S-transferase was found to be normal and the activity of catalase and glutathione peroxidase was found to increase. Pretreatment with SkQ1 normalized the activity of pro-oxidant enzymes NADPH-oxidase and myeloperoxidase, which was significantly higher in hyperoxia [77].

The activation of Nrf2 can be also mediated by additional signal transduction pathways, e.g. ERK, c-Jun amino-terminal kinase (JNK), AMP-activated protein kinase (AMPK) or PI3K/AKT promoting anti-oxidative effects, which mediate enhanced resistance to oxidative/nitrosative stress as well as to further oxidative insults [78, 79]. Constitutive stabilization of Nrf2 is found in several human cancers [80–82] and is associated with increased cancer chemotherapy resistance, enhanced tumor progression [83, 84] and poor prognosis and/or survival for patients [81, 85, 86]. Mechanisms by which the Nrf2 signaling pathway is constitutively activated in several types of cancer include *(1)* somatic mutations of Keap1 disrupting the binding capacity to Nrf2, *(2)* epigenetic silencing of Keap1 and *(3)* transcriptional induction of Nrf2 by oncogenes such as K-ras, B-raf or c-myc [87]. Furthermore, increased levels of hydrogen peroxide and increased Nrf2 activity in tumor cells, result in an enhanced anaerobic glycolysis and utilization of the pentose phosphate pathway activity to generate NAD(P)H equivalents necessary for the Trx and GSH-based antioxidative systems [88, 89]. Since NAD(P)H generating enzymes are Nrf2 targets, the energy metabolism is directly connected with the redox homeostasis. This is confirmed by an increased metabolic oxidative/nitrosative stress and cytotoxicity in response to the inhibition of glycolysis and/or the pentose phosphate pathways in combination with an inhibition of the Trx metabolism [90]. In contrast, knock down of Nrf2 suppresses tumor growth, inhibits cell proliferation and promotes increased apoptosis [85, 91]. The fact, that several cancers exhibit induced Nrf2 levels associated with enhanced tumor progression and chemotherapy resistance, whereas the lack of Nrf2 has opposite effects, Nrf2 represents a promising target for cancer therapies.

### Activation of NF-kB transcription factor by ROS/RNS

Since its discovery in 1986, NF-kB (nuclear factor kappa B) transcription factor has aroused a wide interest in its unusual regulation, diverse stimuli that activate it, and its apparent involvement in a variety of human diseases, including atherosclerosis, asthma, diabetes, cancer, arthritis, AIDS, inflammatory diseases, etc. NF-kB belongs to the REL-family of pluriprotein transcription activators. It is a regulatory protein that controls the expression of numerous inducible and tissue-specific NF-kB responsible genes and participates in the regulation of pro-inflammatory and immune cellular responses, the regulation of cell proliferation, and apoptosis [92–94].

The NF-kB/REL family of transcription factors consist of homo- and heterodimers of five distinct proteins, the REL subfamily proteins (p65/RELA, RELB, and c-REL) and the NF-kB subfamily proteins (p50, and p52, and its precursors p105 and p100, respectively). All NF-kB/REL proteins contain a Ref-1-homology domain (RHD), which is responsible for dimerization, recognition and binding to DNA as well as interaction with the inhibitory kB (IkB) proteins. The IkB family is composed of IkB-α, IkB-β, IkB-ϵ, IkB-γ, and BCL-3, all of which possess typical ankyrin repeats that bind to the RHD of NF-kB/REL proteins to interfere with their function. The IkB-kinase complex (IKK complex) catalyzes phosphorylation of IkBs with the result that IkBs are targeted for degradation by the 26S proteasome thereby freeing NF-kB. The latter then binds to the DNA consensus sequence 5′-GGGRNNYYCC-3′ (R is a purine, Y is a pyrimidine, and N is any base) in the promoter/enhancer regions of target genes [95].

Activation of IKK occurs also by phosphorylation and is catalyzed by an IKK kinase, including TGF-β-activated kinase (TAK1) or NF-kB inducing kinase-1 (NIK1), all of which can be regulated by hydrogen peroxide [96]. Alternatively, the reduced form of the dynein light chain protein LC8 binds to IkB and inhibits its phosphorylation by IKKs. Hydrogen peroxide induces dimerization of LC8 by a disulfide bond, promoting dissociation from IkB, and NF-kB activation [97]. On the other hand, inhibition of activation of NF-kB by hydrogen peroxide can be mediated by Keap1-dependent degradation of IKKβ [98].

NF-kB target genes mainly include enzymes involved in the antioxidant response such as ferritin heavy chain [99] and SOD2 [100]. Another NF-kB target gene that contributes to both survival and innate immune functions is the HIF-1α gene, encoding the oxygen-regulated subunit of the hypoxia responsive transcription factor HIF-1 [101]. On the other hand, hydroquinone and tert-butyl hydroquinone, prototypes of phenolic antioxidants, block lipopolysaccharide-induced transcription of TNFα and NF-kB as they block the formation of NF-kB/DNA binding complexes [102].

NF-kB activation is stimulated by pro-oxidative cell status, especially by an increased presence of hydrogen peroxide. The exact signaling cascade seems to be due to the activation of MAP kinase pathway. On the other side, the activation of NF-kB is blocked by thiol components such as N-acetyl-L-cysteine (glutathione precursor) and antioxidants. It has been demonstrated that a low concentration of thiol compounds in the cell, primarily glutathione as the most widespread thiol compound, plays a key role in positive regulation of NF-kB activity [103–108]. Therefore, the mechanisms that regulate and control the level of glutathione in the cell indirectly participate in regulating the expression of the genes with an NF-kB binding site in the promoter. It is postulated that ROS/RNS regulate NF-kB activity and modify some of the links in a complex activating cascade of NF-kB transcription factor: *(1)* oxidation of key sites in enzymes (kinases) that phosphorylate and activate IkB kinase which, due to the phosphorylation of serine residues of inhibitors, activates NF-kB complex; *(2)* redox modulation of IkB kinase activity; and *(3)* modulation of transport of the activated NF-kB from the cytoplasm into the nucleus. Since NF-kB has a ubiquitous role in controlling cytokine activity and immunoregulatory genes, the inhibition of NF-kB activity by steroid hormones, antioxidants, non-steroid anti-inflammatory drugs, and protease inhibitors represents a pharmacological basis for the intervening adjuvant therapy in numerous diseases, including cancer, diabetes mellitus, AIDS, and diverse inflammatory disorders [109].

### Activation of AP-1 transcription factor by ROS/RNS

The mechanism for activating AP-1 (activator protein 1) transcription factor by free radicals is one of the best-explained mechanisms. AP-1 is a family of dimeric bZIP transcription factors including Jun, Fos, ATF/CREB, JDP and Maf that usually form heterodimers that bind to a TPA-responsive element (TRE, 5′-TGAG/CTCA-3′) or cAMP response elements (CRE, 5′-TGACGTCA-3′). They regulate several cellular processes, including cell proliferation, apoptosis, survival, and differentiation. AP-1, when upregulated, concentrates in the nucleus to activate gene expression [110]. Hydrogen peroxide was shown to induce transcription of both c-Jun and c-Fos via activating the JNK, p38 MAPK and ERK signaling cascades [111], while antioxidants like butylated hydroxyanisole (BHA) and pyrrolidine dithiocarbamate (PDTC) induce AP-1 binding activity and AP-1-dependent gene expression including glutathione S-transferase [112]. The AP-1 activity is regulated not only at the genetic level (regulation of transcription) but also at both posttranscriptional and post-translational levels [113]. The exposure of HeLa cells to hydrogen peroxide or UV radiation leads to a significant increase in DNK-binding activity of AP-1, irrespective of Fos and Jun protein synthesis. Under the conditions, AP-1 is activated by phosphorylation of specific residues of AP-1 subunits. For example, the activation of cascade phosphorylation of MAP kinase family (c-Jun N-terminal kinase [JNK] i.e. stress-activated protein kinase [SAPK]) leads to the phosphorylation of two serine residues (Ser-63 and Ser- 73) in the Jun subunit of AP-1 to promote the activation of this subunit [113]. Oxidative/nitrosative stress, too, can be a factor that mediates promotion of ligand effects exerted at the posttranslational level of AP-1 activity regulation, by activating signaling via JNK protein kinases. Fos protein in AP-1 is also activated, by the phosphorylation of threonine residue (Thr-232) due to fos-regulatory kinase activated by p21ras protein [113]. On the other hand, the phosphorylation of Jun protein (Thr-231, Ser-243, and Ser-149) by constitutive protein kinases, casein kinases II, and DNK-dependent protein kinase results in inhibiting the binding of AP-1 to DNK. Dephosphorylation of threonine and serine residues of Jun protein increases the affinity of AP-1 active transcription factor for binding to DNK. This transcription factor is activated due to PKC that initiates dephosphorylation of the Jun subunit of AP-1 protein following activation of phosphatases. P21ras itself is a signaling target of radicals generated by hydrogen peroxide and nitric oxide. Their overexpression is also responsible for the activation of PKC and dephosphorylation of serine and threonine residues in DNA binding domain of c-Jun [114, 115]. Dimer complex (Fos and Jun products) interacts with DNA regulatory element known as AP-1 binding site or with CRE. These elements are present in the regulatory domain of AP-1 inducible genes [116].

Although several other pathways can be regulated by ROS/RNS, the primary responses against different intensities of intermediate oxidative/nitrosative stress are mainly modulated by the cooperation of the three pathways: Nf-kB, AP1 and MAP kinases. However, with increasing oxidative/nitrosative stress, the Nrf2/Keap pathway is also needed to induce antioxidant defenses and to minimize oxidative/nitrosative damage. When these defenses are not strong enough to contend against oxidative/nitrosative stress, the cell induces formation/opening of the mitochondrial permeability transition pore, as an efficient way to decrease ROS/RNS production, by decreasing the mitochondrial membrane mitochondrial potential. However, this increases the oxidative state in the cell, and resulting in a homeostatic disruption of the redox balance, cell damage, and apoptosis [117, 118].

### Activation of miRNAs by ROS/RNS

miRNAs are short strands of noncoding RNA that posttranscriptionally regulate gene expression and are being considered key elements in the pathogenesis of various disease [119]. The level of miRNAs can be modulated at the transcription and/or processing level by stress-induced factors like p53 or NF-kB [120]. There are current studies indicating that the miRNAs expression can be sensitive to the presence of intracellular hydrogen peroxide levels. Epigenetic regulation at the DNA level is an important mechanism involved in hydrogen peroxide-mediated expression changes of multiple genes, indicating that miRNA expression is very sensitive to hydrogen peroxide stimulation [121]. For example, in smooth muscle cells, the cellular treatment with hydrogen peroxide resulted in an upregulation of microRNA-21 [121]. In addition, the expression of miR-181a in hydrogen peroxide-treated H9c2 cells (cell line derived from rat heart tissue was markedly upregulated [122]. In that context, miRNAs could be modulating intracellular pathways formed by the participation of multiple proteins. That would be the case of ROS/RNS-mediated events [122]. Furthermore, the relationship of mitochondrial dysfunction, defined as the result of the increased production of ROS/RNS in mitochondria, the accumulation of mitochondrial DNA damage, and the progressive respiratory chain alteration, plus the altered expression of miRNA is now being established for the onset of some diseases [123]. Unraveling the signaling events initiated at the cellular level by oxidative/nitrosative free radicals, as well as the changes that occur in microRNA expression, is important not only because of the need to better understand the disease pathogenesis, but also because of its implications in the search for new biomarkers and the design of new therapeutic targets [124].

## Redox sensitive regulation of gene expression mediated by protein tyrosine phosphatases and protein kinases

ROS/RNS have emerged as important modulators of intracellular transduction signaling. These radicals interact with redox-sensitive signaling molecules including protein tyrosine phosphatases, protein kinases and ion channels, that contain cysteine residues whose SH groups are oxidized, causing a change in their biological activity, regulating cellular processes like growth factor signaling, hypoxic signal transduction, autophagy, immune responses, and stem cell proliferation and differentiation [125, 126]. These redox changes include activation/deactivation cycles of redox metabolism and expression of antioxidant enzymes expression that are essential to maintain nucleofilic tone and oxidant/antioxidant balance in the cells [127].

### Protein tyrosine phosphatases

All tyrosine phosphatases have a conserved 230-amino acid domain that contains a reactive and redox-regulated cysteine, which catalyzes the hydrolysis of protein phosphotyrosine residues by the formation of a cysteinyl-phosphate intermediate, that later is hydrolyzed by an activated water molecule. Because of the unique environment of the tyrosine phosphatase active site, the catalytic cysteine presents an unusually low pKa value and is thus deprotonated at physiological pH, existing as a thiolate anion (Cys–S−). Oxidation of this residue to sulfenic acid by hydrogen peroxide renders the tyrosine phosphatases inactive. This oxidation of cysteine to sulfenic acid is reversible, while oxidation by the addition of two (sulfinic acid) or three (sulfonic acid) oxygens to the active site cysteine is irreversible. These modifications can be further stabilized by the formation of inter or intramolecular disulfide (S–S) or sulfenyl–amide bonds. Thus ROS/RNS significantly inhibit activity of tyrosine phosphatases, resulting in increased tyrosine phosphorylation [128].

### Protein kinases

Protein kinases are a group of enzymes that phosphorylate tyrosine, threonine, and/or serine residues of target proteins altering their function. Susceptible cysteines in some serine/theonine protein kinases are directly modified by ROS/RNS. Protein kinase C (PKC) contains a cysteine rich domain susceptible to oxidation, while oxidation of Cys-245 and Cys-487 in the kinase domain of the nonreceptor tyrosine kinase Src results in the activation of the protein [129]. Mitogen-activated protein kinases (MAPKs) are a family of serine/threonine kinases that play a central role in coupling various extracellular signals to a variety of biological processes, such as gene expression, cell proliferation, differentiation, and cell death. The activity of MAP kinases (ERK, c-Jun and p38) is regulated by phosphorylation cascades: MAPKs activation is induced through the phosphorylation of their threonyl and tyrosyl residues within a tripeptide motif TXY by a dual specificity kinase termed MAP kinase kinase (MKK), which in turn is phosphorylated and activated by an upstream kinase called MAPK kinase kinase (MAPKKK) [130]. However, MAPKs can also be activated by ROS/RNS.

Many growth factor and cytokine receptors bearing cysteine-rich motifs can be targets of oxidative/nitrosative stress. Thus, insulin-like growth factor-I (IGF-I) activates ERK pathway through ROS/RNS-mediated activation of EGF receptor, which by mediation of Ras-GTPase activates Raf and MEK, phosphorylating ERK [131, 132]. ROS/RNS may also activate MAPK pathways through the oxidative modification of the intracellular kinases. ASK-1, a member of the MAP3K superfamily for JNK and p38, binds to reduced thioredoxin in nonstressed cells. Upon oxidative/nitrosative stress, thioredoxin becomes oxidized and dissociates from ASK-1, leading to activation of JNK and p38 pathways through oligomerization of ASK-1 [133]. Another mechanism for MAPK activation by ROS/RNS includes the inactivation and degradation of the MAPK phosphatases (MPKs). Son et al. reported that hydrogen peroxide inactivates MKPs by oxidation of their catalytic cysteine, which leads to sustained activation of the MAPK pathway [134]. However, Zhou et al. found that upregulation of MKP-1 expression by hydrogen peroxide correlates with inactivation of JNK and p38 activity [135]. This paradox in the roles of ROS/RNS as “inducers” in the regulation of MKP-1 expression and as “inhibitors” may be, at least in part, related to differences in the concentrations of ROS/RNS and of the redox sensitivity of Cys residues within MAPK phosphatases. Big mitogen-activated protein kinase-1 (BMK-1), also known as Erk5, is a MAPK identified in 1995. Similar to other three MAPKs, BMK-1 has been shown to be activated various extracellular stimuli such as epidermal growth factor, IL-6, and hypoxia and regulated by MAPK cascade. As a member of MAPK family, BMK-1 has also been linked to various cellular events including proliferation, migration, and apoptosis [136].

The activity of several complexes of the electron transport chain is modulated by post-translational modifications such as S-nitrosylation, S-glutathionylation or electrophile additions. Complex I (NADH ubiquinone oxidoreductase) is modified by nitric oxide or its derivatives and glutathione [137, 138]. The S-nitrosylation of complex I correlates with a significant loss of activity that is reversed by thiol reductants. S-nitrosylation was also associated with increased superoxide production from complex I. The fact that mitochondrial superoxide formation can be regulated by S-nitrosylation of complex I may play an important role in mitochondrial redox signalling. Complex I has two transitional states, the active (A) and the deactive (D) states, and the complex is S-nitrosylated in the D state [139]. The A to D transition may take place during hypoxia and this might be important in the setting of ischemia–reperfusion. Other studies have reported nitrotyrosine modification on the complex [140]. In addition, reversible glutathionylation of complex I increases mitochondrial superoxide production [138]. Other electron transport complexes, in particular, complex II (succinate dehydrogenase) and complex V (ATP synthase), have been shown to be modified by reactive species. Likewise, mitochondrial matrix proteins such as NADP + -isocitrate dehydrogenase, α-ketoglutarate dehydrogenase and aconitase, as well as proteins of the intermembrane space such as creatine kinase and cytochrome c can also be modified by reactive species, affecting in most cases their catalytic activity. In response to stress or to an increased demand in energy, mitochondria produce a limited amount of ROS/RNS that act as signaling molecules, initiating a molecular stress response that leads to transcriptional changes in the nucleus [141, 142]. A transient ROS/RNS signal generates an endogenous response that allows detoxification of ROS/RNS by inducing defence enzymes such as superoxide dismutase or catalase, as well as other stress defence pathways. This process is called retrograde response. The release of ROS/RNS from mitochondria can function in numerous signalling pathways. Some examples are the regulation of cytosolic stress kinases, modulation of hypoxic signalling, and activation of macroautophagy [143–145].

In contrast to this function of ROS/RNS as signalling molecules, sustained high levels of ROS/RNS can cause intracellular damage. This nonlinear response to mitochondrial ROS/RNS is known as mitochondrial hormesis or mitohormesis [146]. This concept considers ROS/RNS as essential signalling molecules, and not just harmful and damaging by-products of mitochondrial metabolism.

## Antioxidants

**Fig. 3:**
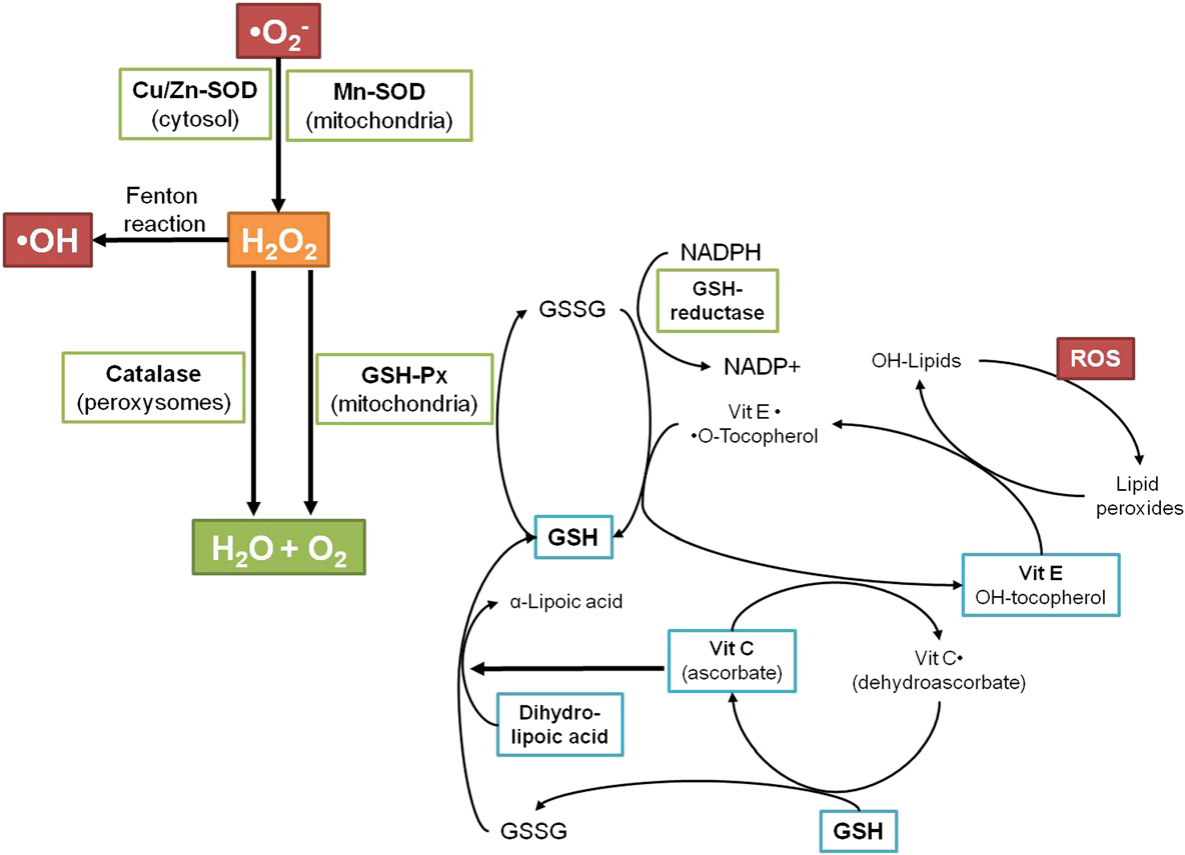
Antioxidant defenses in the organism

An antioxidant substance in the cell is present at low concentrations and significantly reduces or prevents oxidation of the oxidizable substrate. Humans have developed highly complex antioxidant systems (enzymatic and non-enzymatic), which work synergistically, and together with each other to protect the cells and organ systems of the body against free radical damage (Fig. 3). The antioxidants can endogenous or obtained exogenously as a part of a diet or as dietary supplements. Some dietary compounds that do not neutralize free radicals, but enhance endogenous activity may also be classified as antioxidants. An ideal antioxidant should be readily absorbed and eliminate free radicals, and chelate redox metals at physiologically suitable levels. It should also work in both aqueous and/or membrane domains and effect gene expression in a positive way. Endogenous antioxidants play a critical role in keeping optimal cellular functions and thus systemic health and well-being. However, under conditions, which support oxidative/nitrosative stress, endogenous antioxidants may not be sufficient, and dietary antioxidants may be required to maintain optimal cellular functions. The most efficient enzymatic antioxidants contain glutathione peroxidase, catalase and superoxide dismutase. Non-enzymatic antioxidants include Vitamin E and C, thiol antioxidants (glutathione, thioredoxin and lipoic acid), melatonin, carotenoids, natural flavonoids, and other compounds. Some antioxidants can interact with other antioxidants regenerating their original properties; this mechanism is usually referred to as the “antioxidant network”. There is growing evidence to support a link between increased levels of ROS/RNS and deteriorated activities of enzymatic and nonenzymatic antioxidants in various diseases [2, 147].

### Enzymatic antioxidants

#### Glutathione peroxidase

Glutathione peroxidases catalyse the oxidation of glutathione at direction of a hydroperoxide, which may be hydrogen peroxide or another species such as a lipid hydroperoxide:

$$ \mathrm{RO}\mathrm{O}\mathrm{H} + 2\mathrm{G}\mathrm{S}\mathrm{H}\to \mathrm{GSSG} + {\mathrm{H}}_2\mathrm{O} + \mathrm{R}\mathrm{O}\mathrm{H} $$

Other peroxides, including lipid hydroperoxides, can also act as substrates for these enzymes, which may hence play a role in repairing damage resulting from lipid peroxidation. There are two forms of this enzyme, one which is selenium-dependent (GPx, EC1.11.1.19) and the other, which is selenium-independent (glutathione-S-transferase, GST, EC 2.5.1.18). The differences rise from the number of subunits, catalytic mechanism, and the bonding of selenium at the active centre, and glutathione metabolism is one of the most important antioxidative defense mechanisms present in the cells. There are four different Se-dependent glutathione peroxidases present in humans, and these are known to add two electrons to reduce peroxides by forming selenole (Se-OH) and the antioxidant properties of these seleno-enzymes allow them to eliminate peroxides as potential substrates for the Fenton reaction. Selenium-dependent glutathione peroxidase acts in association with tripeptide glutathione (GSH), which is exist in high concentrations in cells and catalyzes the conversion of hydrogen peroxide or organic peroxide to water or alcohol while simultaneously oxidizing GSH. It also competes with catalase for hydrogen peroxide as a substrate and is the major source of protection against low levels of oxidative/nitrosative stress [148].

#### Catalase

Catalase (EC 1.11.1.6) was the first antioxidant enzyme to be characterized and catalyses conversion of hydrogen peroxide to water and oxygen:

$$ {\mathrm{H}}_2{\mathrm{O}}_2\to 2{\mathrm{H}}_2\mathrm{O} + {\mathrm{O}}_2 $$

Catalase consists of four subunits, each containing a haem group and a molecule of NADPH. The rate constant for the reactions described above is extremely high (~10^7^ M/sec), implying that it is virtually impossible to saturate the enzyme in vivo. This enzyme is present in the peroxisome of aerobic cells and is very efficient in promoting the conversion of hydrogen peroxide to water and molecular oxygen. Catalase has one of the highest turnover rates for all enzymes: one molecule of catalase can convert approximately 6 million molecules of hydrogen peroxide to water and oxygen each minute. The greatest activity is present in liver and erythrocytes but some catalase is found in all tissues [125].

#### Superoxide dismutase

Superoxide dismutase (EC 1.15.1.1) is one of the most potent intracellular enzymatic antioxidants and it catalyzes the conversion of superoxide anions to dioxygen and hydrogen peroxide:

$$ {{\mathrm{O}}_2}^{-} + {{\mathrm{O}}_2}^{-} + 2{\mathrm{H}}^{+}\to {\mathrm{H}}_2{\mathrm{O}}_2 + {\mathrm{O}}_2 $$

The hydrogen peroxide is removed by catalase or glutathione peroxidase, as described above. Superoxide dismutase exists in several isoforms, which differ in the nature of active metal centre, amino acid composition, co-factors and other features. There are three forms of SOD present in humans: cytosolic Cu, Zn-SOD, mitochondrial Mn-SOD, and extracellular-SOD. Superoxide dismutase neutralizes superoxide ions by going through successive oxidative and reductive cycles of transition metal ions at its active site. This enzyme has two similar subunits and each of the subunit includes as the active site, a dinuclear metal cluster constituted by copper and zinc ions, and it specifically catalyzes the dismutation of the superoxide anion to oxygen and water. The mitochondrial Mn-SOD is a homotetramer with a molecular weight of 96 kDa and includes one manganese atom per subunit, and it cycles from Mn(III) to Mn(II), and back to Mn(III) during the two-step dismutation of superoxide anion. Extracellular superoxide dismutase contains copper and zinc, and is a tetrameric secretary glycoprotein having a high affinity for certain glycosaminoglycans [149].

### Nonenzymatic antioxidants

#### Vitamin E

**Fig. 4:**
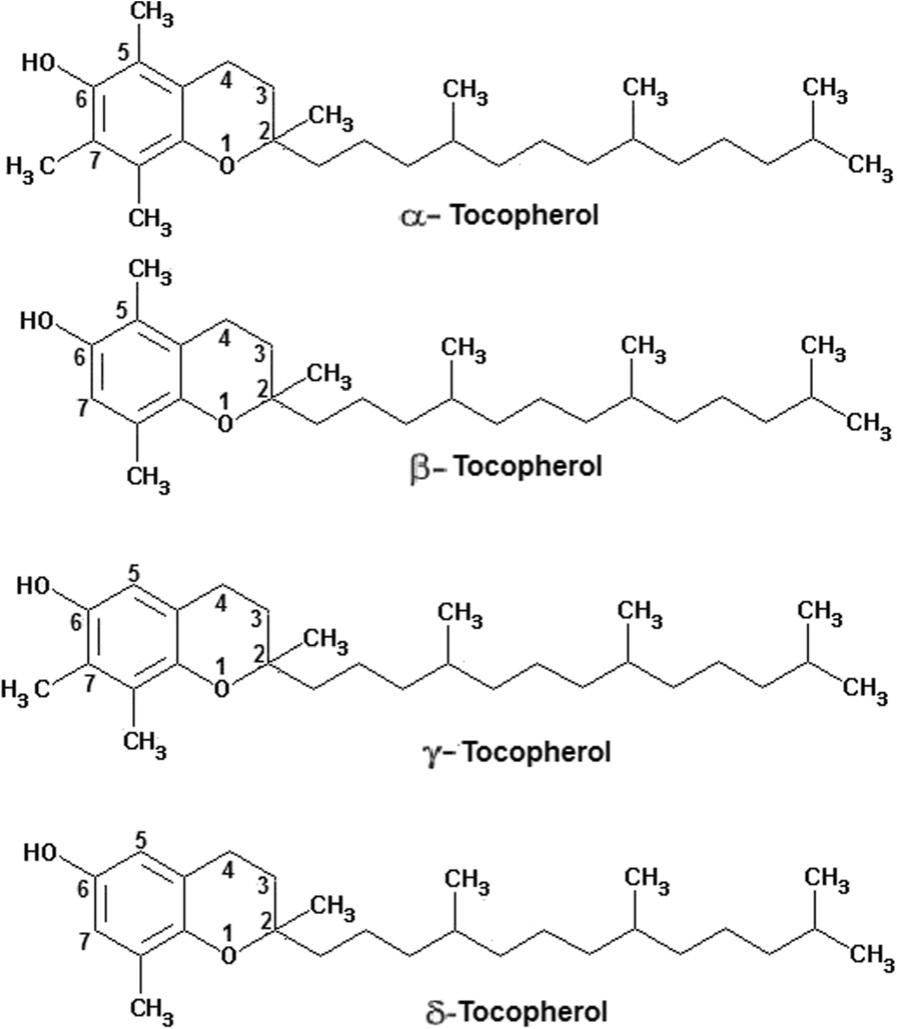
Chemical structure of the tocopherols

This is a fat-soluble vitamin existing in eight different forms [150]. The tocopherols (α, β, γ, and δ) have a chromanol ring and a phytyl tail, and differ in the number and position of the methyl groups on the ring (Fig. 4). These compounds are lipid soluble and have pronounced antioxidant properties. They react more rapidly than polyunsaturated fatty acids with peroxyl radicals and hence act to break the chain reaction of lipid peroxidation [151]. In addition to its antioxidant role, vitamin E might also have a structural role in stabilising membranes. Vitamin E deficiency is rare in humans, although it might cause haemolysis and might contribute to the peripheral neuropathy that occurs in abetalipoproteinaemia. In cell membranes and lipoproteins the essential antioxidant function of vitamin E is to trap peroxyl radicals and to break the chain reaction of lipid peroxidation. Vitamin E doesn’t prevent the initial formation of carbon centred radicals in a lipid rich environment, but does minimise the formation of secondary radicals. α-Tocopherol is the most effective antioxidant of the tocopherols and is also the plentiful in humans. It quickly reacts with a peroxyl radical to form a relatively stable tocopheroxyl radical, with the excess charge associated with the extra electron being dispersed across the chromanol ring. This resonance stabilised radical might subsequently react in one of several ways. α-Tocopherol might be regenerated by reaction at the aqueous interface with ascorbate or another aqueous phase chain breaking antioxidant, such as reduced glutathione or urate. As another option, two α-tocopheroxyl radicals may combine to form a stable dimer, or the radical may be completely oxidised to form tocopherol quinone. The main function of Vitamin E is to protect against lipid peroxidation, and there is also evidence to suggest that α-tocopherol and ascorbic acid function together in a cyclic-type of process. During the antioxidant reaction, α-tocopherol is converted to an α-tocopherol radical by the donation of a variable hydrogen to a lipid or lipid peroxyl radical, and the α-tocopherol radical may hence be reduced to the original α-tocopherol form by ascorbic acid [152].

#### Vitamin C (ascorbic acid)

**Fig. 5:**
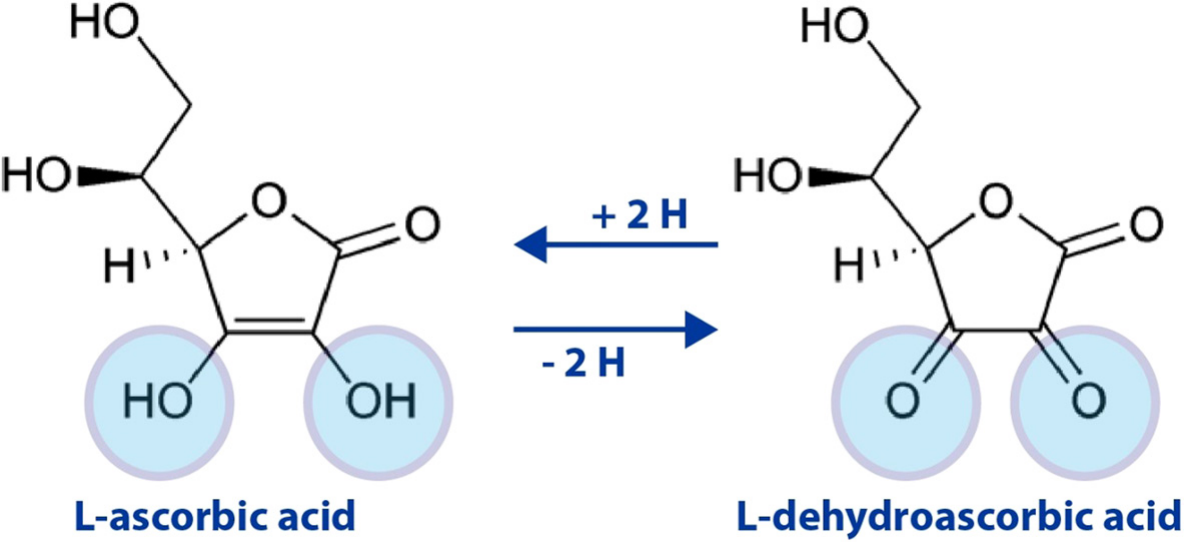
The oxidation-reduction (redox) reaction of vitamin C, molecular forms in equilibrium. L-dehydroascorbic acid also possesses biological activity, due to that in the body it is reduced to form ascorbic acid

This is an important antioxidant and thus works in aqueous environments of the body. Furthermore, ascorbic acid can be oxidized in the extracellular environment in the presence of metal ions to dehydroascorbic acid, which is transported into the cell through the glucose transporter (Fig. 5). Its primary antioxidant partners are Vitamin E and the carotenoids as well as working alone with the antioxidant enzymes. Vitamin C cooperating with Vitamin E to regenerate α-tocopherol from α-tocopherol radicals in membranes and lipoproteins, and also raises glutathione levels in the cell, thus it is playing an important role in protein thiol group protection against oxidation. In cells, it is maintained in its reduced form by reaction with glutathione, which catalyzes by protein disulfide isomerase and glutaredoxins. Vitamin C is a reducing agent and can reduce and thereby neutralize, ROS such as hydrogen peroxide [153].

#### Thiol antioxidants

##### Glutathione

The most thiol antioxidant is the tripeptide glutathione (GSH), which is a multifunctional intracellular antioxidant. It is noticed to be the major thiol-disulphide redox buffer of the cell. It is abundant in cytosol, nuclei, and mitochondria, and is the major soluble antioxidant in these cell compartments. Also, it is considered to be playing a role in cell senescence since studies involving human fibroblasts have shown that the intracellular glutathione level has an important influence on the induction of a post-mitotic phenotype, and that by implication depletion of glutathione plays a significant role in the cellular aging in human skin. The reduced form of glutathione is GSH, glutathione, while the oxidized form is GSSG, glutathione disulphide. The antioxidant capacity of thiol compounds is due to the sulphur atom, which can easily accommodate the loss of a single electron. Oxidized glutathione (GSSG) is accumulated inside the cells and the ratio of GSH/GSSG is a good measure of oxidative stress of an organism. The protective roles of glutathione against oxidative/nitrosative stress are that it can act as a co-factor for several detoxifying enzymes, participate in amino acid transport across plasma membrane, scavenge hydroxyl radical and singlet oxygen directly, and regenerate Vitamins C and E back to their active forms [154]. It has been well established that a decrease in GSH concentration may be associated with aging and the pathogenesis of many diseases, including rheumatoid arthritis, AIDS, alcoholic liver disease, cataract genesis, respiratory distress syndrome, cardiovascular disease, and Werner syndrome. Furthermore, there is a drastic depletion in cytoplasmic concentrations of GSH within the substantia nigra of patients with Parkinson. Depletion of total GSH (GSH + 2 GSSG + protein-bound glutathione) and a decreased GSH:GSSG ratio are indicators of oxidative stress in ischemic brain disease, cardiovascular diseases, and cancer, and decreased concentrations of GSH are consistently observed in both types of diabetes mellitus [155–159].

##### Thioredoxin

Another thiol antioxidant is the thioredoxin (TRX) system; these are proteins with oxidoreductase activity and are universal in both mammalian and prokaryotic cells. It also contains a disulphide and possesses two redox-active cysteins within a conserved active site (Cys-Gly-Pro-Cys). Thioredoxin contains two adjacent –SH groups in its reduced form that are converted to a disulphide unit in oxidized TRX when it undergoes redox reactions with multiple proteins. Thioredoxin levels are much less than GSH, however, TRX and GSH may have overlapping as well as compartmentalized functions in the activation and regulation of transcription factors. Secretion of thioredoxin under conditions of oxidative/nitrosative stress and inflammation has been observed from many normal or neoplastic cells [160, 161]. Clinically, plasma thioredoxin levels are raised in diseases associated with oxidative/nitrosative stress such as arthritis or HIV infection, arrhythmia, ischemia reperfusion injury, and hypertension [116, 162, 163].

##### α-Lipoic acid (1,2-dithione-3-pentanoic acid)

**Fig. 6:**
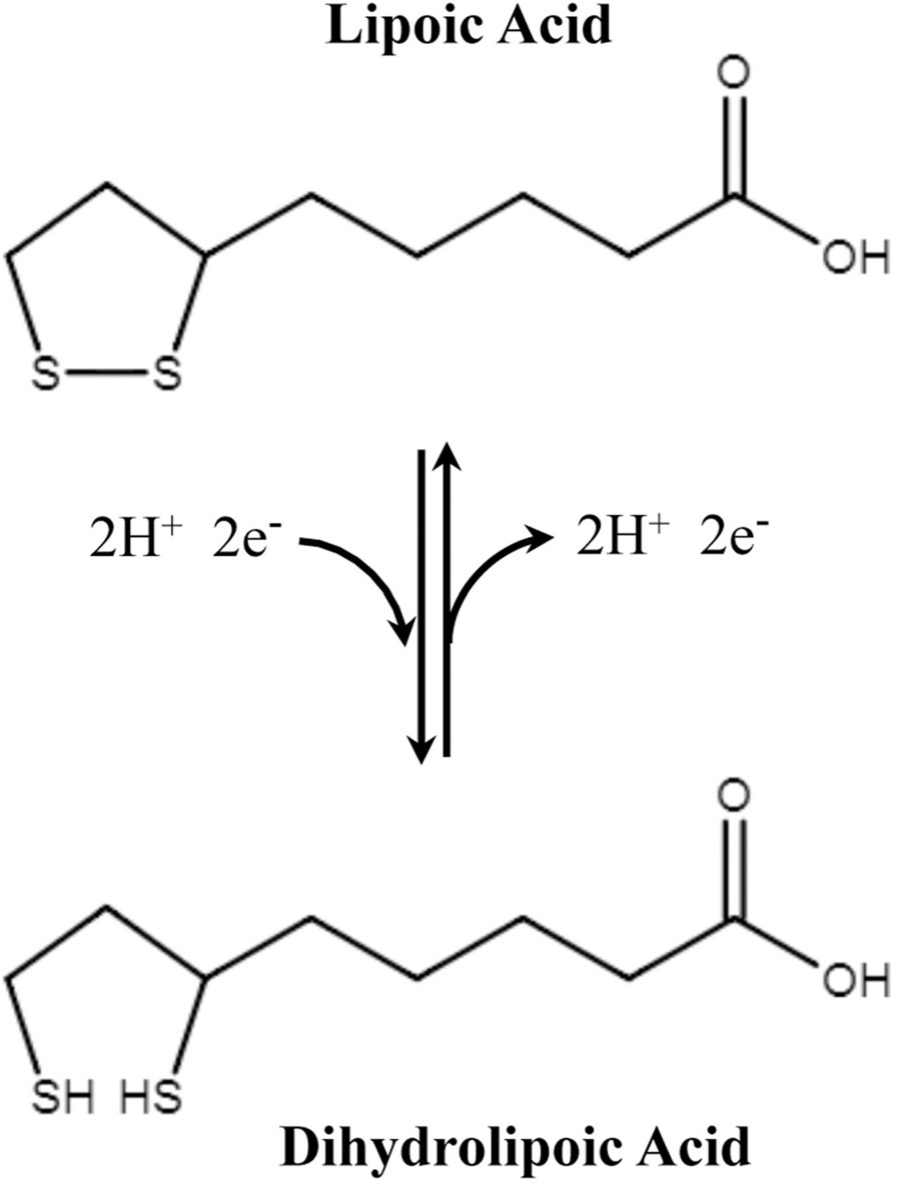
Oxidized and reduced forms of lipoic acid

The third valuable thiol antioxidant is the natural compound α-Lipoic acid (ALA), which is a sulfur-containing antioxidant with metal-chelating and antiglycation capabilities. In contrast to many antioxidants, which are active only in lipid or aqueous phase, lipoic acid is active in both lipid and aqueous phases. Lipoic acid (LA) is readily digested, absorbed and is rapidly converted to Dihydrolipoic acid (DHLA) by NADH or NADPH in most tissues (Fig. 6). Researches have demonstrated superior antioxidant activity of DHLA as compared to LA. Since DHLA neutralizes free radicals it is known to regenerate Vitamin C which is even better than GSH and Vitamin E from their oxidized forms. DHLA has metal chelating properties which help the body to get rid of accumulated ingested toxins. It has been shown previously that oxidants lead to cell death via lysosomal breaking away and that this latter event may involve intralysosomal iron which catalyzes Fenton-type chemistry and resultant peroxidative damage to lysosomal membranes. LA stabilize lysosomes against oxidative/nitrosative stress, perhaps by chelating intralysosomal iron and, consequently, preventing intralysosomal Fenton reactions. Packer et al. proposed a hypothesis of LA inducing cystine/cysteine uptake which investigated the role of LA in stimulating GSH biosynthesis [164]. As an antioxidant, LA directly terminates free radicals, chelates transition metal ions (e.g., iron and copper), increases cytosolic glutathione and vitamin C levels and prevents toxicities associated with their loss [165]. Exogenous administration of LA has been found to have therapeutic potential in neurodegenerative disorders also. Furthermore, LA crosss the blood-brain barrier and is enclosed by all areas of the central and peripheral nervous system. Lipid peroxides (LPO) are biomarkers of free radical-associated oxidative stress. Free radical attack on poly unsaturated fatty acids (PUFA) in the biological system is thought to produce a sequence of reactions, which lead to the formation of both conjugated dienes and lipid hydroperoxides [166]. Hence the possible mechanisms for the protecting effects of LA against oxidative stress may be as follows: *(a)* LA can be reduced to dihydrolipoic acid by NADH, *(b)* DHLA is a potent antioxidant to scavenge excess oxidants, and recycle other antioxidants such as vitamin E, C and glutathione, *(c)* DHLA chelate metals to prevent free radical generation, thus to diminish oxidant attacks on bio-macromolecules, *(d)* LA is the key co-factor of pyruvate dehydrogenase and α-ketoglutarate dehydrogenase the enzymes sensitive to oxidative stress, *(e)* supplementation of sufficient LA can stimulate activities of enzymes, thereby promoting and ameliorating oxidative phosphorylation and mitochondrial respiration and *(f)* LA promotes the antioxidant defense by inducing phase two enzymes, such as glutathione synthetase to increase antioxidant GSH.

##### N-acetylcysteine

**Fig. 7:**
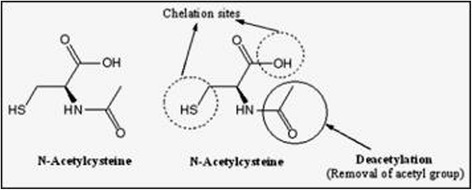
Structure of N-acetyl cysteine (NAC) depicting (1) two chelating sites (thiol and hydroxyl) and (2) deacetylation responsible for its antioxidant potential due to the generation of glutathione

N-acetyl-L-cysteine (NAC), is a thiol containing antioxidant that has been used to decrease conditions of oxidative/nitrosative stress. It reduces liver damage caused by paracetamol over dosage in human, and attenuates liver damage and prevents liver and plasma GSH depletion in mice [167]. Furthermore, NAC is generally used for the treatment of acetaminophen-induced hepatotoxicity, although it has a drawback as it must be given within 8 h after acetaminophen intoxication, and it can also cause other side-effects including vomiting, nausea, and even shock. Therefore, the need for alternative, more effective, and widely applicable antidotes for acetaminophen-induced liver injury is warranted [168, 169]. Its antioxidant action is considered to originate from its ability to stimulate GSH synthesis, therefore maintaining intracellular GSH levels and scavenging ROS/RNS. NAC, is quickly deacetylated to cysteine and thus may increase GSH levels by providing the substrate for the rate limiting step in GSH synthesis. Structure of NAC along with possible chelating sites is presented in Fig. 7. NAC is known to have metal-chelating properties and has been used in several clinical conditions. Thiol groups present in NAC act to decrease free radical and provide chelating site for metals. Thus, NAC has a potent ability to renovate the impaired prooxidant antioxidant balance in metal poisoning and various diseases [170].

##### Melatonin

**Fig. 8:**
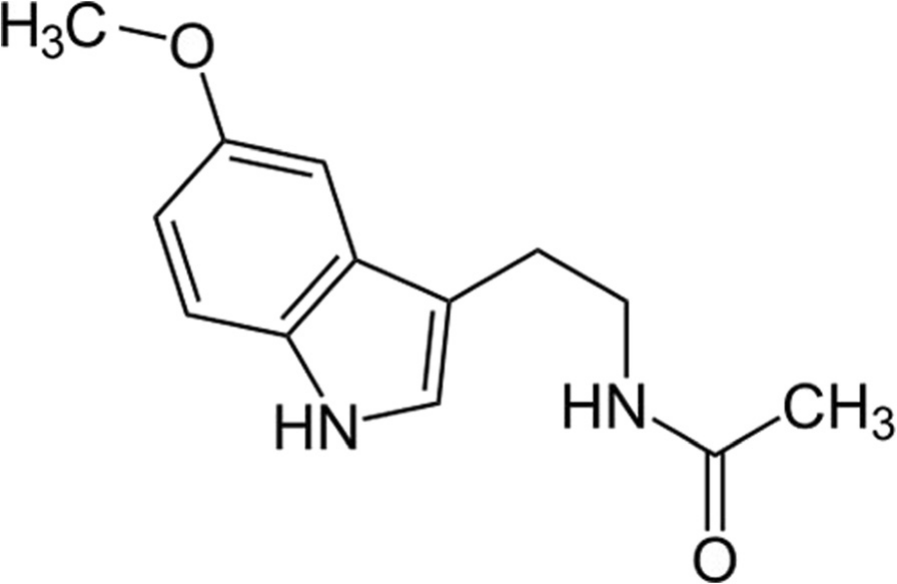
Chemical structure of melatonin

Melatonin (N-acetyl-5-methoxytryptamine) is a chief secretory product of the pineal gland in the brain which is well known for its functional versatility. In hundreds of investigations, melatonin has been documented as a direct free radical scavenger and an indirect antioxidant, as well as an important immunomodulatory agent (Fig. 8). It is believed that the radical scavenging ability of melatonin works via electron donoration to remove a variety of ROS/RNS, including the highly toxic hydroxyl radical. Furthermore, melatonin stimulates a number of antioxidative enzymes including superoxide dismutase, glutathione peroxidase, glutathione reductase, and catalase. Additionally, melatonin experimentally enhances intracellular glutathione (another important antioxidant) levels by stimulating the rate-limiting enzyme in its synthesis, gamma-glutamylcysteine synthase. Melatonin also inhibits the pro-oxidant enzymes such as nitric oxide synthase, xanthine oxidase and lipoxygenase. Finally, there is evidence that melatonin stabilizes cellular membranes, thereby probably helping them resist oxidative damage. Most recently, melatonin has been shown to increase the efficiency of the electron transport chain and, as a consequence, to reduce electron leakage and the generation of free radicals. These multiple actions make melatonin a potentially useful agent in the treatment of neurological disorders that have oxidative damage as part of their etiological basis [171].

##### Carotenoids

**Fig. 9:**
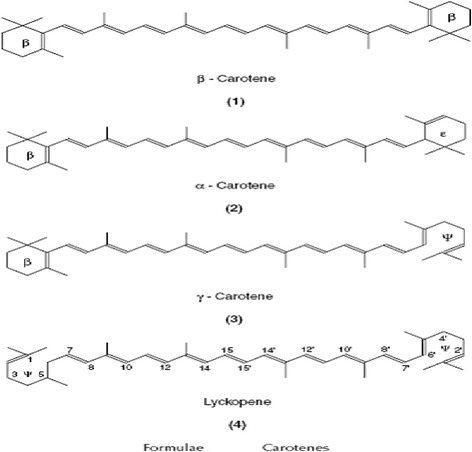
Chemical structure of selected carotenoids

The carotenoids are a group of lipid soluble antioxidants which based around an isoprenoid carbon skeleton. The most important of these is α-carotene, although these present in membranes and lipoproteins. They are particularly efficient scavengers of singlet oxygen, but can also trap peroxyl radicals at low oxygen pressure with an efficiency at least as major as that of α-tocopherol. Because these conditions prevail in many biological tissues, the carotenoids play a role in preventing in vivo lipid peroxidation. Carotenoids with bring are characterized with pro-vitamin A activity; the highest activity is represented by β-carotene because it contains two brings on both ends of the carbon chain (Fig. 9). Vitamin A also has antioxidant properties, which do not, but, show any dependency on oxygen concentration [172].

##### Flavonoids

**Fig. 10:**
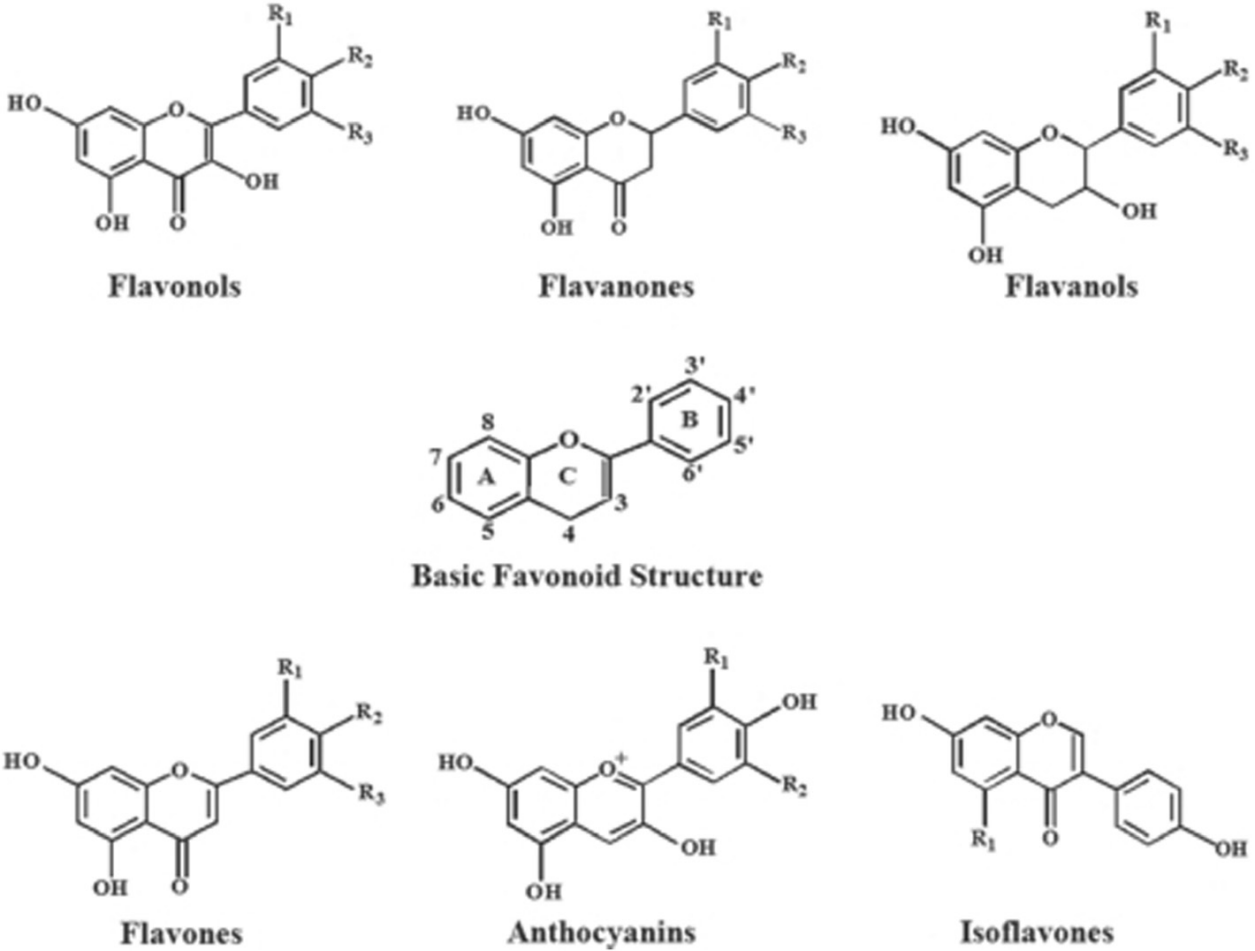
Chemical structure of some flavonoids

These are a broad class of low molecular ubiquitous groups of plant metabolites and are an integral part of the human diet. Flavonoids are benzo-γ-pyrone derivatives consisting of phenolic and pyrane rings and during metabolism hydroxyl groups are added, methylated, sulfated or glucuronidated (Fig. 10). There is intense interest in flavonoids due to their antioxidant and chelating properties and their possible role in the prevention of chronic diseases. Flavonoids are present in food mainly as glycosides and polymers and these comprise a substantial fraction of dietary flavonoids. The biological properties of flavonoids are determined by the extent, nature, and position of the substituents and the number of hydroxyl groups. These factors also determine whether a flavonoid acts as an antioxidant or as a modulator of enzyme activity, or whether it has antimutagenic or cytotoxic properties. The most reported activity of flavonoids is their protection against oxidative/nitrosative stress. Thus flavonoids can scavenge peroxyl radicals, and are effective inhibitors of lipid peroxidation, and can chelate redox-active metals, and therefore prevent catalytic breakdown of hydrogen peroxide (Fenton chemistry). On the other hand, under certain conditions, flavonoids can also display pro-oxidant activity and this is thought to be directly proportional to the total number of hydroxyl groups, and they have also been reported to modulate cell signaling [173].

## Antioxidant supplementation

The last 60 years have been characterized by the understanding of the impact of nutrition and dietary patterns on health. An important part of the population is exposed to the risk of trace element and vitamin deficiency for multiple reasons (e.g., changes in eating habits in many countries, lower food concentration of micronutrients due to intensive agricultural techniques). Children, young women and elders are the most exposed. Antioxidants balances the cell-damaging effects of free radicals. Furthermore, people eat fruits and vegetables, which happen to be good sources of antioxidants, have a lower risk for various diseases such as heart, neurological diseases and there is evidence that some types of vegetables and fruits in general, protect against a number of cancers [174–177]. However, this hypothesis has now been tested in many clinical trials and does not seem to be true, since antioxidant supplements have no clear effect on the risk of chronic diseases such as cancer, diabetes mellitus and heart disease. Though cells are equipped with an effective repertoire of antioxidant enzymes as well as small antioxidant molecules, these substances can not be sufficient enough to normalize the redox status during oxidative/nitrosative stress. Under such conditions supplementation with exogenous antioxidants is required to provide the redox homeostasis in cells. Since several plant products are rich in antioxidants and micronutrients, it is possibly that dietary antioxidant supplementation protects against the oxidative/nitrosative stress mediated disease development. Therefore, antioxidant supplementation has become an increasingly popular practice to maintain optimal body function [178–182].

Much debate has arisen about whether antioxidant supplementation alters the efficacy of cancer chemotherapy. Some have argued that antioxidants scavenge the ROS/RNS integral to the activity of certain chemotherapy drugs, thereby diminishing treatment efficacy. Others suggest antioxidants may mitigate toxicity and thus allow for uninterrupted treatment schedules and a reduced need for lowering chemotherapy doses. One of the main mechanisms of chemotherapy drugs against cancer cells is the formation of ROS/RNS. Drugs with free radical mechanisms include but are not limited to alkylating agents (e.g., melphalan, cyclophosphamide), anthracyclines (e.g., doxorubicin, epirubicin), podophyllin derivatives (e.g., etoposide), platinum coordination complexes (e.g., cisplatin, carboplatin) and camptothecins (e.g. topotecan, irinotecan) [183]. Unfortunately, these ROS/RNS often are the source of serious side effects, as well [184]. None of the trials reported evidence of significant decreases in efficacy from antioxidant supplementation during chemotherapy. Many of the studies indicated that antioxidant supplementation resulted in either increased survival times, increased tumor responses, or both, as well as fewer toxicities than controls [185]. Studies are now attempting to develop new antioxidants either of natural or synthetic origin. In the last 2 decades, there has been great progress in the development of biomarkers of oxidative/nitrosative stress that may eventually be useful in disease prevention. The challenges for the future are *(1)* to validate available biomarkers for oxidative/nitrosative damage in animal and human studies based on their specificity, stability for storage, reproducibility, causal relation with disease, and response to antioxidant intervention; *(2)* to examine the basal levels of oxidative/nitrosative damage in healthy subjects; and *(3)* to assess the long-term effect of antioxidants on oxidative/nitrosative damage by well designed, randomized, controlled trials in humans and as well as to examine the consistency of the findings among various studies. The biomarkers of oxidative/nitrosative damage, if validated, may open the way for the development of early detection and prevention strategies for oxidative/nitrosative stress-associated human diseases. The successful development of effective antioxidant therapies remains a key goal, the attainment of which is required to elucidate the role played by accumulation of (toxic) oxidized molecules in the clinical picture of diseases associated with oxidative/nitrosative stress. The use of biomarkers provides a logical scientific basis for major intervention trials of antioxidants; such trials could, in turn, eventually validate or disprove the biomarker concept. Any intervention trial that does take place should be accompanied by measurements of one or more relevant biomarkers at intervals during the study. If the endpoint of the trial is disease incidence or mortality, such studies could help to validate or disprove the biomarker concept. However, with regard to this matter, we must highlight the disappointing clinical evidence showing the failure of antioxidant therapy for oxidative/nitrosative stress-associated pathologies such as cardiovascular disease, coronary artery disease, and neurodegenerative diseases [179–186].

## Limitations of antioxidant supplementation

The primary worry concerning antioxidant supplementation is their potentially deleterious effects on ROS/RNS production especially when precise modulation of ROS/RNS levels are needed to allow normal cell function. The removal of too many ROS/RNS and their derived products by antioxidant supplementation may upset the cell signaling pathways and actually increase the risk of chronic disease. It should be pointed out, however, that physiologically necessary signaling mediator is produced under controlled and regulated manner and reacts with sensing molecules in a fine‑tuned, specific, and regulated manner. On the contrary, free radical-mediated lipid peroxidation proceeds randomly without specificity. Lipid peroxidation can neither be programmed nor regulated. This has raised a question if removal of too many ROS/RNS by supplementation of antioxidants may upset the cell signaling pathways and actually increase the risk of chronic diseases [187, 188]. Furthermore, some negative effects of antioxidants when used in dietary supplements (ascorbic acid, flavanoids, carotenoids, α-lipoic acid and synthetic compounds) have came out in the last few decades [189]. For example, Ascorbic acid has both antioxidant and pro-oxidant effects, depending upon the dose [190]. Low electron potential and resonance stability of ascorbate and the ascorbyl radical have enabled ascorbic acid to enjoy the privilege as an antioxidant [191, 192]. In ascorbic acid alone treated rats, ascorbic acid has been found to act as a CYP inhibitor. Similar activity has also been observed for other antioxidants-quercetin and chitosan oligosaccahrides [193], which may act as potential CYP inhibitors. Specifically, Phase I genes of xenobiotic biotransformation, namely, CYP1A1, CYP2E1, and CYP2C29, have been previously reported to be downregulated in female rats in the presence of a well known antioxidant, resveratrol [194]. The in vivo pro-oxidant/antioxidant activity of beta-carotene and lycopene has also been found to depend on their interaction with biological membranes and the other co-antioxidant molecules like vitamin C or E [195]. At higher oxygen tension, carotenoids tend to lose their effectiveness as antioxidants. In a turn around to this, the pro-oxidant effect of low levels of tocopherol is evident at low oxygen tension [196]. Moreover, α-lipoic acid exerts a protective effect on the kidney of diabetic rats but a prooxidant effect in nondiabetic animals [189]. The pro-oxidant effects have been attributed to dehydroxylipoic acid (DHLA), the reduced metabolite of α-lipoic acid owing to its ability to reduce iron, initiate reactive sulfur-containing radicals, and thus damage proteins such as alpha 1-antiproteinase and creatine kinase playing a role in renal homeostasis [189]. An increase in α-lipoic acid and DHLA-induced mitochondrial and submitochondrial production in rat liver and NADPH-induced and expression of p47phox in the nondiabetic kidney has also been observed [197].

Depending on the type and level of ROS and RNS, duration of exposure, antioxidant status of tissues, exposure to free radicals and their metabolites leads to different responses—increased proliferation, interrupted cell cycle, apoptosis, or necrosis [165].

## Conclusion

Protection from the influence of ROS/RNS being an important issue has become the attraction of the scientists in recent years to understand the mechanism of action of various antioxidants. There has been ever increasing knowledge in the role of oxygen derived pro-oxidants and antioxidants that play crucial role in both normal metabolism and several clinical disease states. Antioxidants exhibit pro-oxidant activity depending on the specific set of conditions. Of particular importance are their dosage and redox conditions in the cell. Furthermore, while antioxidants may have reduced free-radical damage to normal tissues leading to diminished toxicity, the non-oxidative cytotoxic mechanisms of the drugs may remain unaffected by antioxidant supplementation. Further, the significant reductions in toxicity may alleviate dose-limiting toxicities to such an extent that more patients successfully complete prescribed regimens. Advances in the field of biochemistry including enzymology have led to the use of various enzymes as well as endogenous and exogenous antioxidants having low molecular weight that can inhibit the harmful effect of oxidants.

## Abbreviations

ROS, Reactive Oxygen Species; RNS, Reactive Nitrogen Species; NOS, Nitric Oxide Synthase; NADPH, Reduced Nicotinamide dinucleotide phosphate; MDA, Malondialdehyde; HNE, 4-hydroxy-2-nonenal; F_2_-IsoPs, isoprostanes; Se-OH, selenole; SOD, Superoxide Dismutase; GSH, Reduced Glutathione; GSSG, Oxidized glutathione; TRX, thioredoxin; LA, Lipoic acid; DHLA, Dihydrolipoic acid; NADH, Nicotinamide dinucleotide; LPO, Lipid peroxides; PUFA, polyunsaturated fatty acids; NAC, N-acetyl-L-cysteine; N-acetyl-5-methoxytryptamine, Melatonin; NF-kB, nuclear factor kappa B; Keap1, Kelch-like ECH-associated protein 1; ARE, Antioxidant responsive element; Nrf2, Nuclear factor erythroid-derived 2-related factor 2; AP-1, Activator protein 1; HO-1, Heme oxygenase-1; TXNRD1, thioredoxin reductase-1; CYP, Cytochrome P450; SkQ1, 10-(6′-plastoquinonyl) decyl-triphenyl-phosphonium; RHD, Ref-1-homology domain; IKK complex, IkB-kinase complex; TAK1, TGF-β-activated kinase; NIK1, NF-kB inducing kinase-1; BHA, butylated hydroxyanisole; PDTC, pyrrolidine dithiocarbamate; CRE, cAMP-responsible element; IGF-I, insulin-like growth factor-I; BMK-1, Big mitogen-activated protein kinase-1; JNK, c-Jun amino-terminal kinase; AMPK, AMP-activated protein kinase; MAPKs, Mitogen-activated protein kinases
